# Duration of Dual Antiplatelet Therapy after Percutaneous Coronary Intervention of Unprotected Left Main Coronary Artery Stenosis: 6 versus 12 Months

**DOI:** 10.3390/jcm13185449

**Published:** 2024-09-13

**Authors:** Tau Sarra Hartikainen, Sina Mertins, Max Behrens, Franz-Josef Neumann, Christian Marc Valina, Nikolaus Löffelhardt, Faridun Daniel Rahimi Nedjat, Philipp Breitbart, Kilian Franke, Dirk Westermann, Miroslaw Ferenc

**Affiliations:** 1 Department of Cardiology and Angiology, University Heart Center Freiburg—Bad Krozingen, 79189 Bad Krozingen, Germany; tau.hartikainen@uniklinik-freiburg.de (T.S.H.); franz-josef.neumann@uniklinik-freiburg.de (F.-J.N.); christian.valina@uniklinik-freiburg.de (C.M.V.); nikolaus.loeffelhardt@uniklinik-freiburg.de (N.L.); faridun.rahimi@uniklinik-freiburg.de (F.D.R.N.); philipp.breitbart@uniklinik-freiburg.de (P.B.); kilian.franke@uniklinik-freiburg.de (K.F.); dirk.westermann@uniklinik-freiburg.de (D.W.); miroslaw.ferenc@uniklinik-freiburg.de (M.F.); 2 Institute of Medical Biometry and Statistics, Faculty of Medicine and Medical Center, University of Freiburg, 79104 Freiburg, Germany; max.behrens@uniklinik-freiburg.de; 3 Institute of Heart Diseases, Wroclaw Medical University, 50-345 Wroclaw, Poland

**Keywords:** percutaneous coronary intervention, left main disease, dual antiplatelet therapy, coronary artery disease, drug-eluting stents

## Abstract

**Background/Objectives**: For patients with percutaneous coronary intervention (PCI) of an unprotected left main coronary artery (uLMCA) stenosis, the optimal duration of dual antiplatelet therapy (DAPT) remains a matter of debate. The purpose of this study was to compare clinical outcomes of 6- versus 12-month DAPT duration in patients with PCI of an uLMCA and stable angina. **Methods**: In this retrospective analysis, we included consecutive patients of our centre who underwent PCI of uLMCA stenosis for stable angina and who received DAPT with acetylsalicylic acid and clopidogrel for either 6 or 12 months. The primary endpoint was the composite of all-cause mortality, myocardial infarction, and target lesion revascularization at one year. Secondary endpoints included individual components of the primary endpoint, definite/probable stent thrombosis, and bleeding. Clinical outcomes were assessed by unadjusted analysis and by inverse probability of treatment weighting (IPTW). **Results**: Out of 984 included patients, 339 (34.5%) received DAPT for 6 months and 645 (65.5%) for 12 months. The primary endpoint occurred in 51 patients (15.2%) in the 6-month group and in 104 (16.3%) in the 12-month group (*p* = 0.674). Incidences of stent thrombosis (0.9% versus 0.3%, *p* = 0.224) and BARC 3,4,5 bleeding (6% versus 5.8%, *p* = 0.808) were also comparable in both groups. We found no significant differences in the primary endpoint and its components or BARC 3,4,5 bleeding between 6 and 12 months. **Conclusions**: Our findings do not support the extension of DAPT beyond 6 months after PCI for uLMCA in patients with stable angina.

## 1. Introduction

A significant stenosis of the left main coronary artery (LMCA) is present in approximately 3–5% of patients with coronary artery disease (CAD) [1]. When left untreated, an LMCA stenosis of greater than 50% is associated with poor outcomes [2]. Treatment options for LMCA stenosis are either surgical revascularization or percutaneous coronary intervention (PCI). The latter less invasive therapeutic strategy has been increasingly performed thanks to improvements in stent technology, advancements in the expertise of interventional cardiologists, and encouraging results of randomized clinical trials [2,3].

In patients with CAD and without indication for oral anticoagulation, dual antiplatelet therapy (DAPT) consisting of acetylsalicylic acid (ASA) and a P2Y_12_ receptor inhibitor is established as the standard therapy after PCI in order to prevent thrombotic events [2]. Regarding the optimal DAPT duration, it is a challenge to balance ischemic and bleeding risks. The optimal DAPT duration has been investigated in several large, randomized trials [4,5]. Based on these trials and according to the recommendations of the European Society of Cardiology (ESC) guidelines, DAPT consisting of ASA and clopidogrel is advised for a duration of 6 months in patients with stable coronary artery disease (SCAD) undergoing stent implantation [2]. In special circumstances, a shortened or extended DAPT duration may be considered [2].

Limited data exist on the optimal duration of DAPT after implantation of drug-eluting stents (DESs) in patients suffering from unprotected left main coronary artery (uLMCA) stenosis. Therefore, the purpose of this retrospective study was to investigate clinical outcomes depending on 6 versus 12 months of DAPT duration after PCI of uLMCA.

## 2. Materials and Methods

### 2.1. Study Design and Patient Population

**Figure 1:**
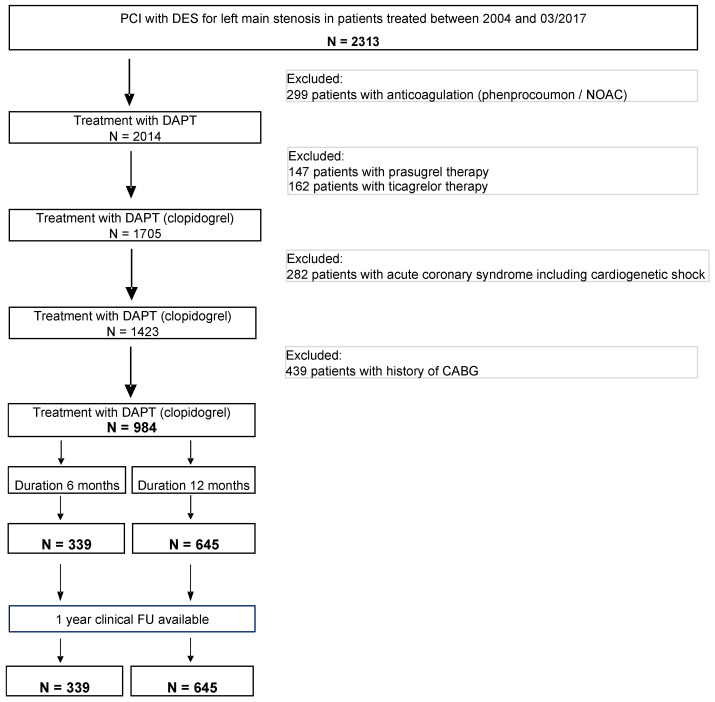
Flow diagram of inclusion and exclusion criteria in the Bad Krozingen Left Main Registry.

The study was designed as a single-centre, retrospective analysis from the Left Main Registry of the University Heart Center Freiburg—Bad Krozingen. Patients who underwent PCI with DES implantation of a de novo left main stenosis with a diameter of >50% were consecutively enrolled in the period from January 2004 to March 2017. The included patients either presented with stable preprocedural angina pectoris or had a positive myocardial stress test prior to the index PCI. All patients received postprocedural DAPT which contained the P2Y_12_ receptor inhibitor clopidogrel in addition to ASA. Patients treated with other P2Y_12_ inhibitors like prasugrel or ticagrelor, as well as those with established indication for extended DAPT, such as acute coronary syndromes, were excluded from this analysis. Further exclusion criteria were the indication for an oral anticoagulation and cardiogenic shock at the time of index PCI (Figure 1). Baseline demographic, clinical, angiographic, and procedural characteristics were collected in the registry and all patients were followed up to assess for clinical endpoints compiled one year after index PCI. The Left Main Registry of the University Heart Center Freiburg—Bad Krozingen was approved by the local ethics committee and was conducted in accordance with the Declaration of Helsinki.

### 2.2. Procedural Aspects

Stenoses of the LMCA were categorized in either ostial, shaft, or distal lesions. The degree of calcification in the treated lesions was visually estimated and classified as none, minor, moderate, or severe calcification. Distal LMCA bifurcation lesions were categorized according to the Medina classification by visual estimation [6].

Patients without continuous intake of ASA received a loading dose of 150–300 mg orally or 75–150 mg intravenously before the PCI and a lifelong maintenance dose of 100 mg ASA was recommended. All included patients further received a loading dose of 600 mg clopidogrel. The maintenance daily dose of clopidogrel was 75 mg. The choice of DAPT duration of either 6 or 12 months after PCI was left to the operator’s discretion and was based on the patients’ individual risks of ischemia and bleeding.

Treatment of uLMCA bifurcation stenosis was performed according to the provisional side-branch stenting strategy [7,8]. In upfront two-stent techniques, Culotte stenting or the T and small protrusion (TAP) technique were applied [9]. The selection of the implanted DES type was as indicated by the treating interventionalist. Intravascular imaging was performed as per operator preference. During the study period, this occurred in a minority of PCIs.

### 2.3. Follow-Up and Study Endpoints

The follow-up protocol included contact by mailed questionnaires or telephone calls at 30 days and at 1 year. Referring cardiologists and/or general practitioners were contacted for further information in case of reported events.

The primary composite endpoint was defined as a major adverse cardiac event (MACE) at one year consisting of all-cause mortality, incident myocardial infarction (MI) and target lesion revascularization (TLR). The secondary endpoint consisted of the individual components from the primary combined endpoint as well as bleeding complications and stent thrombosis at one year. The diagnosis of myocardial infarction was made according to the fourth universal definition [10]. TLR and stent thrombosis were defined as recommended by the Academic Research Consortium (ARC) [11]. Bleeding events were classified by the Bleeding Academic Research Consortium (BARC) criteria [12]. To ensure comparability with major contemporary studies, we chose the BARC criteria, which were developed specifically for bleeding after PCI and are the preferred tool for reporting bleeding in interventional cardiology studies. Our key bleeding endpoint was the composite of BARC 3, 4, and 5. We also analyzed BARC 2 bleeding. A summary of the definitions of the BARC criteria can be found in Table S1 in the Supplementary Materials.

### 2.4. Statistical Methods

**Figure 2:**
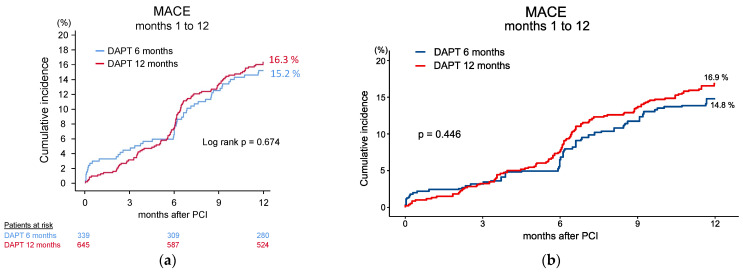

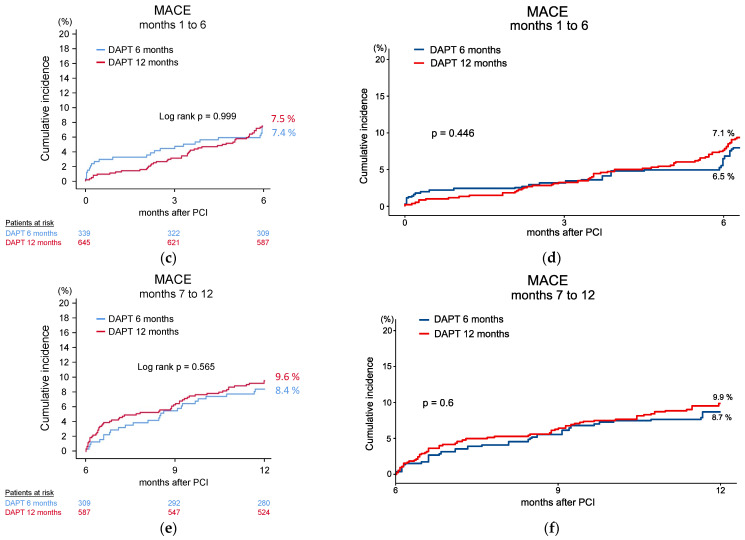
Unadjusted (**a**,**c**,**e**) and IPTW-adjusted (**b**,**d**,**f**) time-to-event curves for the primary endpoint during the entire follow-up (**a**,**b**) and according to landmark analysis for months 1 to 6 (**c**,**d**) and months 7 to 12 (**e**,**f**). Abbreviations: IPTW, inverse probability of treatment weighting; MACEs, major adverse cardiac events; DAPT, dual antiplatelet therapy.

Binary variables are shown as absolute numbers and percentages and were compared with the chi-squared test. Continuous variables are shown as mean and standard deviation for normally distributed data or as median (interquartile range) for non-normally distributed data and compared using the *t*-test or the Mann–Whitney-U test, respectively. The normality of distributions was assessed by the Shapiro–Wilk test. The Kaplan–Meier method was applied for producing survival curves regarding the primary and secondary endpoints and the log rank test was used to test for survival curve differences. We performed unadjusted and adjusted analyses of the endpoints. As the proportional hazards assumption for Cox models was violated with intersecting survival curves (Figure 2), Cox models were not appropriate for multivariable analysis. We, therefore, chose Inverse-Probability of Treatment Weighting (IPTW) to correct for differences in baseline characteristics [13]. The variables included in these analyses were selected prospectively on the basis of their putative relationship with treatment choice or prognosis. Information regarding these variables can be found in Figure S1 in the Supplementary Materials. A *p* value < 0.05 was considered statistically significant.

## 3. Results

### 3.1. Baseline Demographics

In the period from January 2004 to March 2017, we identified 984 patients with uLMCA stenosis undergoing PCI with DES implantation, who met the inclusion criteria. Out of all included patients, 339 (34.5%) received DAPT for a duration of 6 months and 645 (65.5%) for 12 months. The majority of all patients were male, with 256 (75.5%) and 491 (76.1%) in the 6- and 12-month groups, respectively. The median age was 71 years (interquartile range (IQR) 64–78) and 70 years (IQR 61–77) in these groups. Other baseline clinical and demographic characteristics of the study population are displayed in Table 1.

### 3.2. Angiographic and Procedural Characteristics

The majority of the treated uLMCA stenoses were distal lesions (74.9% and 80.6% in the 6- and 12-month groups respectively) with mostly a minor (50.7% and 47.8%) or moderate (31.6% and 32.7%) degree of calcification. True distal left main bifurcation lesions were significantly more common in the 12-month group than in the 6-month group (47.3% vs. 40.4%, *p* = 0.043). Bifurcation lesions were mostly treated with a single-stent strategy (65.8% and 66.4% in the 6- and 12-month group respectively). In cases of a two-stent strategy, TAP stenting was notably more common in both groups (95.7% and 85.7%) as compared to a culotte technique. However, a TAP stenting strategy was significantly more pronounced in the 6-month arm (*p* = 0.005). The used total contrast volume and radiation exposure were both significantly higher in the 6-month group, while fluoroscopy time was comparable in both groups. Table 2 further summarizes angiographic and procedural characteristics.

### 3.3. Clinical Outcomes during Entire Follow-Up

**Figure 3:**
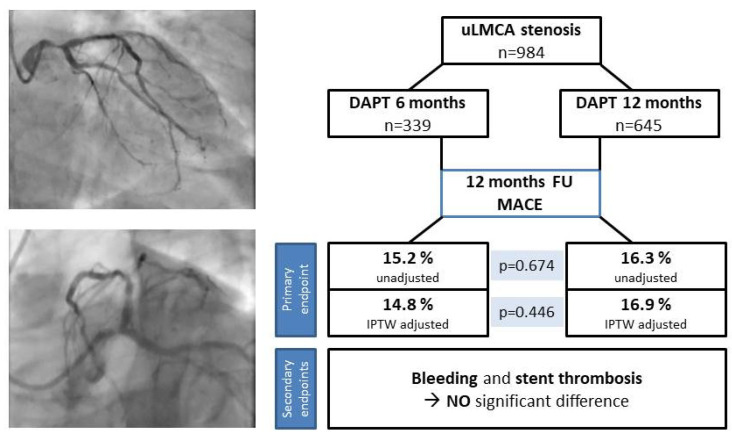
Study plan and clinical outcomes. After 12 months, there was no significant difference regarding the primary composite endpoint (MACE) consisting of all-cause mortality, incident MI, or TLR between the two investigated groups in both unadjusted and adjusted analyses. The rate of bleedings and stent thromboses was similar, as well. Abbreviations: uLMCA, unprotected left main coronary artery; DAPT, dual antiplatelet therapy; MACE, major adverse cardiac event(s); IPTW, inverse probability of treatment weighting; MI, myocardial infarction; TLR, target lesion revascularisation.

The primary composite endpoint MACE occurred in 51 (15.2%) versus 104 (16.3%) patients in the 6- and 12-month group in one year, respectively (*p* = 0.674) (Figure 3, Figure 2a, Table 3). After IPTW adjustment, there were no meaningful differences in the distribution of baseline clinical characteristics between the two groups (Supplemental Figure S1). With this adjustment, there was still no significant difference regarding the primary endpoint between the two groups (*p* = 0.446) (Figure 3, Figure 2b, Table 4). In the secondary endpoint analyses, the individual components of the primary endpoint did not show significant differences, either (Table 3, Supplemental Figures S2–S4). Clinically relevant bleeding events (BARC 3,4,5) occurred in 19 (6%) patients of the 6-month group and in 34 (5.8%) patients of the 12-month group (*p* = 0.808) (Table 3). Likewise, there was no significant difference in BARC 2 bleeding between the two groups (Table 3).

### 3.4. Landmark Analyses

Figure 2c–f show the unadjusted and IPTW-adjusted landmark analysis for the primary endpoint. During the time period when clopidogrel had been discontinued in one of the groups, which was months 7 to 12, there was no appreciable difference in the primary point between the two study groups (Figure 2e,f).

The same was true for the IPTW-adjusted analysis of components of the primary endpoint (Table 3 and Table 4, Supplemental Figures S2–S4). Whereas BARC 2 bleeding was more frequent during months 7 to 12 in the 12-month DAPT group than in the 6-month DAPT group (2.5% versus 0.4%, respectively, *p* = 0.031), clinically relevant bleeding events (BARC 3,4,5) occurred at a similar rate in both groups (1.3% versus 1.8%, respectively, *p* = 0.607) (Table 3).

During the initial six months, the two study groups did not differ significantly in any of the outcome variables investigated (Figure 2c,d, Table 3 and Table 4, Supplemental Figures S2–S4).

## 4. Discussion

In this registry-based study of patients undergoing PCI of an uLMCA stenosis for stable coronary artery disease, 6 months of DAPT was not associated with an increased risk of ischaemic events compared with 12 months of DAPT duration. Regardless of DAPT duration, similar rates of MACE and its components were observed in both unadjusted and IPTW-adjusted analyses. Conversely, there was no reduction in the risk of clinically relevant bleeding (BARC 3,4,5) with shorter DAPT. In both groups, clinically relevant bleeding was rare. It was mostly confined to the first 6 months, and thus occurred independently of the DAPT regimen prescribed. There was, however, a signal for less BARC 2 bleeding after discontinuation of clopidogrel.

Evaluation of the baseline characteristics of this study reveals that patients in the 6-month DAPT group had worse systolic function of the left ventricle and higher C-reactive protein concentrations at baseline. During the intervention, the used contrast medium volume and radiation exposure were also both significantly higher in this group. These parameters might reflect that the 6-month group had a worse health status before the intervention and a more complicated PCI. Since this study was not randomized, the treating physicians might have been more prone to choose a shorter duration of DAPT because of these factors. The appropriate method to address this issue is IPTW. The IPTW-adjusted analyses, which included all of the abovementioned characteristics, did not result in an altered outcome between the two investigated groups.

Based on randomized trials and an individual patient data meta-analysis [14], current guidelines on myocardial revascularization support PCI as a reasonable option for the treatment uLMCA in patients with a low anatomical complexity of CAD [2,15]. This has led to an increasing number of patients that receive an interventional therapy of uLMCA disease. Thus, physicians are confronted more frequently with choosing the optimal medical therapy after such an intervention.

After implantation of DES, the prevention of ischemic events due to a stent thrombosis has utmost priority. A dual antiplatelet therapy (DAPT) is the adjunctive medication of choice to prevent such events [2]. At the same time, the risk for bleeding events increases under the intake of a DAPT. Hence, the goal is to balance bleeding and ischemic risks in each patient, since the occurrence of any of these two events has an effect on the outcome and mortality of CAD patients [16].

When choosing the optimal duration of DAPT, several factors should be considered. These include the individual characteristics of the patient including pre-existing comorbidities, whether revascularization was performed in an acute or elective setting, and the type and location of the treated stenosis. A relevant stenosis of the LMCA affects blood flow to a large part of the myocardium. Therefore, the jeopardized myocardial area in case of an ischemic event is larger as compared to more peripheral coronary artery stenoses. It is thought that this is the reason for higher mortality and MACE in patients with LMCA stenosis [17,18]. Thus, a longer duration of DAPT might be useful in this subgroup of patients receiving a PCI.

The current guidelines on myocardial revascularization do not offer specific recommendations regarding DAPT duration after PCI of uLMCA stenosis because, as for now, there is a lack of randomized controlled studies for this subgroup. However, the guidelines encourage the prolongation of DAPT beyond 6 months in selected patients: those with low bleeding but high ischemic risk [2,4]. A meta-analysis of six randomized controlled studies has revealed that especially those with complex interventions of the coronary arteries—for example, an intervention of the LMCA—might profit from a longer DAPT duration [19]. However, this applies primarily for patients with low bleeding risk. In a study by Costa et al., patients with complex lesions only gained a benefit from a prolonged DAPT if they had a low bleeding risk score. Those with high risk of bleeding paradoxically also had an elevated risk of ischemia [20]. This study, however, did not specifically include patients with uLMCA stenosis.

Choi et al. showed in a retrospective analysis of patients with an intervention of a LMCA stenosis that a DAPT duration of more than 12 months as compared to 12 months or less resulted in less cardiovascular events without an increase in bleeding events [21]. Wang et al. validated these results by showing that a prolonged intake of DAPT for more than 12 months was associated with an improved outcome for patients that had not experienced an MACE during the first 12 months of DAPT [22]. Controversially, a posthoc subgroup analysis of the randomized Xience versus Coronary Artery Bypass Surgery for Effectiveness of Left Main Revascularization (EXCEL) trial showed no difference regarding the event-free survival in patients with either a 1- or 3-year DAPT duration [23]. Similarly, the STOPDAPT-2 trial showed lower rates of a combination of cardiovascular and bleeding outcomes in patients with a very short DAPT duration of 1 month compared to a 12-month DAPT duration with ASA and clopidogrel [24]. These results were confirmed in a subgroup analysis for patients who underwent complex PCI [25]. The IDEAL-LM study on the other hand resulted in non-inferiority of a 4-month versus a 12-month duration of DAPT [26]. Since the patients in the IDEAL LM-study also received two different stent types in the compared arms, devolvement of these results into clinical practise is difficult. In summary, the data regarding the optimal duration of DAPT after a LMCA intervention are inconsistent and the varying populations and study designs make an interpretation of the data difficult.

Previous studies have focused on different subgroups, different durations of DAPT, different combinations of antiplatelet drugs and different types of coronary lesions. Our study adds to the existing data by providing information on the real-world performance of either 6 or 12 months DAPT duration in patients with PCI of uLMCA. Our analyses of patients with a PCI of an uLMCA stenosis aimed to explore whether a DAPT duration of 12 months yields an improved outcome compared to the recommended 6 months of DAPT for stable CAD in an all-comer population. We detected no significant difference in the two investigated groups regarding the composite endpoint consisting of all-cause mortality, incident MI, and TLR at 1 year. These results persisted after adjusting for multiple factors by IPTW. Since these analyses were performed in a real-world, all-comer cohort, the included patients might be more representative of the typical patients that receive an interventional treatment of uLMCA than in previous investigations.

## 5. Limitations

When interpreting the results of our study, the following aspects need to be considered. This was a retrospective, non-randomized, single-centre analysis of patients undergoing PCI of an uLMCA stenosis. The choice of the duration of DAPT after the PCI was made by the treating interventionalist, who was aware of all clinical data of the treated patient. To adjust for differences in baseline characteristics and treatment bias, we performed IPTW, which was effective as shown by standardized mean differences well below 0.1 for all variables after weighting (Figure S1 in the Supplementary Materials). Nevertheless, a residual selection bias due to unmeasured confounders cannot be excluded. Despite inclusion of close to thousand patients, our study was not sufficiently powered to conclusively assess rare endpoints, such as stent thrombosis. We only investigated a DAPT duration of either 6 or 12 months. Durations longer than 12 months, as in some of the cited, previous studies, were not included and a long-term follow-up of more than 12 months was not available. We only included patients with stable CAD. Hence, our study cannot inform treatment decisions in patients with uLMCA PCI in acute coronary syndromes; these patients have an established indication for prolonged DAPT [2]. Also, patients with indication for oral anticoagulation were excluded from these analyses. Consistent with current guidelines, clopidogrel was the only P2Y_12_ inhibitor investigated. We did not systematically assess compliance with the recommended treatment. Thus, our findings are based on intention-to-treat analyses and should be interpreted accordingly.

## 6. Impact on Daily Practice

The current study does not support a general strategy of extending DAPT to 12 months after PCI of an uLMCA in patients with SCAD to prevent ischaemic complications. Conversely, for those patients with an extended ischaemic risk, in whom a prolonged DAPT duration is considered, it is reassuring to see that in our study this regimen does not carry a substantial excess risk of bleeding. Thus, our study supports an individual choice of DAPT duration in an effort to balance ischaemic and bleeding risks based on individual patient needs. At present, such decisions remain challenging. Further studies are needed to provide more evidence-based insight into the criteria that inform the optimal choice of DAPT duration after uLMCA PCI.

## Figures and Tables

**Table 1:** Clinical baseline characteristics.

	DAPT 6 Months *n* = 339	DAPT 12 Months *n* = 645	*p* Value
Age in years [Median (IQR)]	71 (64–78)	70 (61–77)	0.092
Male (%)	256 (75.5%)	491 (76.1%)	0.875
Diabetes mellitus (%)	93 (27.4%)	148 (22.9%)	0.138
Current smoker (%)	34 (10.0%)	78 (12.1%)	0.344
Hypertension (%)	287 (84.7%)	521 (80.8%)	0.137
Family history (%)	91 (26.8%)	244 (37.8%)	**0.001**
Cholesterol in mg/dL [Median (IQR)]	183 (152–213)	179 (153–213)	0.964
LDL cholesterol in mg/dL [Median (IQR)]	111 (88–139)	110 (87–139)	0.933
Serum creatinine in mg/dL [Median (IQR)]	1.0 (0.8–1.2)	1.0 (0.8–1.1)	0.128
CRP in mg/dL [Median (IQR)]	0.3 (0.2–0.5)	0.2 (0.1–0.4)	**<0.001**
Hemoglobin in g/dL [Median (IQR)]	14.1 (13.2–15.1)	14.1 (13.1–15.0)	0.751
Thrombocytes × 1000/μL [Median (IQR)]	217 (206–217)	217 (182–244)	0.733
History of MI (%)	76 (22.4%)	117 (18.1%)	0.109
History of PCI (%)	101 (29.8%)	220 (34.1%)	0.175
LVEF (%)			**0.015**
normal	245 (72.3%)	518 (80.3%)	
mildly reduced	54 (15.9%)	76 (11.8%)	
moderately/severely reduced	40 (11.8%)	51 (7.9%)	

Abbreviations: IQR, interquartile range; LDL, low-density lipoprotein; CRP, c-reactive protein; MI, myocardial infarction; PCI, percutaneous coronary intervention; LVEF, left ventricular ejection fraction; DAPT, dual antiplatelet therapy.

**Table 2:** Angiographic and procedural characteristics at baseline.

	DAPT 6 Months *n* = 339	DAPT 12 Months *n* = 645	*p* Value
CAD: Number of vessels affected (%)			0.140
1-vessel	0 (0%)	0 (0%)	
2-vessels	130 (38.3%)	216 (33.5%)	
3-vessels	209 (61.7%)	429 (66.5%)	
Location of the LMCA stenosis (%)			**0.041**
Ostial/shaft	85 (25.1%)	125 (19.4%)	
Distal	254 (74.9%)	520 (80.6%)	
True distal left main bifurcation lesions (Medina 111, 101, 011) (%)	137 (40.4%)	305 (47.3%)	**0.043**
Medina Classification (%)			0.498
Medina 1-1-1	89 (35.0%)	214 (41.2%)	
Medina 1-1-0	75 (29.5%)	121 (23.3%)	
Medina 1-0-1	29 (11.4%)	56 (10.8%)	
Medina 1-0-0	26 (10.2%)	54 (10.4%)	
Medina 0-1-1	19 (7.5%)	35 (6.7%)	
Medina 0-1-0	13 (5.1%)	29 (5.6%)	
Medina 0-0-1	3 (1.2%)	11 (2.1%)	
Grade of calcification in the left main stenosis (%)			**0.031**
None	25 (7.4%)	27 (4.2%)	
Minor	172 (50.7%)	308 (47.8%)	
Moderate	107 (31.6%)	211 (32.7%)	
Severe	35 (10.3%)	99 (15.3%)	
Double stenting technique in bifurcation stenosis (%)			**0.005**
Culotte stenting	5 (4.3%)	31 (14.3%)	
TAP stenting	111 (95.7%)	186 (85.7%)	
First stent in main branch (%)			**<0.0001**
Sirolimus (Cypher)	96 (28.3%)	64 (9.9%)	
Paclitaxel (Taxus)	81 (23.9%)	39 (6.0%)	
Zotarolimus (Resolute, Resolute Integrity)	23 (6.8%)	219 (34.0%)	
Everolimus (Xience, Promus)	139 (41.0%)	323 (50.1%)	
Stent diameter in main branch in mm [Median (IQR)]	3.5 (3.5–4)	4.0 (3.5–4)	**<0.001**
Contrast volume in ml [Median (IQR)]	220 (150–300)	200 (120–250)	**0.002**
Fluoroscopy time in min [Median (IQR)]	18 (10–26)	17 (10–24)	0.115
Radiation exposure in μGym^2^ [Median (IQR)]	6133 (3112–10025)	5000 (2983–7992)	**0.003**
Maximal inflation pressure in main branch in atm [Median (IQR)]	16 (14–18)	16 (12–18)	0.072

Abbreviations: CAD, coronary artery disease; LMCA, left main coronary artery; TAP, T-stenting and small protrusion; IQR, interquartile range; DAPT, dual antiplatelet therapy.

**Table 3:** Unadjusted Kaplan–Meier estimates for primary and secondary endpoints for the entire follow-up and by landmark analysis for months 1 to 6 and months 7 to 12.

	DAPT 6 Months *n* = 339	DAPT 12 Months *n* = 645	*p* Value
MACE (%)	51 (15.2%)	104 (16.3%)	0.674
Death of any cause (%)	18 (5.4%)	23 (3.6%)	0.194
Death or myocardial infarction (%)	20 (5.9%)	30 (4.7%)	0.392
Target lesion revascularization (%)	33 (10.1%)	77 (12.4%)	0.304
Stent thrombosis, any (%)	4 (1.2%)	7 (1.1%)	1.0
Definite/probable stent thrombosis (%)	3 (0.9%)	3 (0.5%)	0.420
Early definite/probable stent thrombosis (%)	3 (0.9%)	2 (0.3%)	0.224
Late definite/probable stent thrombosis (%)	0 (0%)	1 (0.2%)	0.470
Bleedings according to BARC classification (%)			
BARC 3,4,5	19 (6%)	34 (5.8%)	0.808
BARC 2	44 (13.3%)	89 (14.1%)	0.785
BARC 3a	9 (2.8%)	20 (3.4%)	0.712
BARC 3b	6 (1.9%)	6 (1%)	0.252
BARC 3c	2 (0.7%)	3 (0.5%)	0.779
BARC 4	0	4 (0.8%)	0.148
BARC 5	2 (0.7%)	1 (0.2%)	0.238
*Months 1 to 6*			
MACE (%)	25 (7.4%)	48 (7.5%)	0.999
Death of any cause (%)	9 (2.7%)	17 (2.7%)	0.973
Death or myocardial infarction (%)	12 (3.5%)	20 (3.1%)	0.694
Target lesion revascularization (%)	14 (4.2%)	30 (4.8%)	0.712
Stent thrombosis, any (%)	4 (1.2%)	4 (0.6%)	0.350
Definite/probable stent thrombosis (%)	3 (0.9%)	3 (0.5%)	0.416
Early definite/probable stent thrombosis (%)	3 (0.9%)	2 (0.3%)	0.224
Late definite/probable stent thrombosis (%)	0	1 (0.2%)	0.470
Bleedings according to BARC classification (%)			
BARC 3,4,5	14 (4.3%)	27 (4.5%)	0.997
BARC 2	43 (13%)	76 (12%)	0.641
BARC 3a	7 (2.1%)	18 (3%)	0.510
BARC 3b	5 (1.6%)	3 (0.5%)	0.091
BARC 3c	1 (0.4%)	3 (0.5%)	0.707
BARC 4	0	2 (0.4%)	0.308
BARC 5	1 (0.3%)	1 (0.2%)	0.636
*Months 7 to 12*			
MACE (%)	26 (8.4%)	56 (9.6%)	0.565
Death of any cause (%)	9 (1.0%)	6 (2.8%)	**0.036**
Death or myocardial infarction (%)	8 (2.5%)	10 (1.6%)	0.366
Target lesion revascularization (%)	19 (6.2%)	47 (8.0%)	0.306
Stent thrombosis, any (%)	0	3 (0.6%)	0.207
Definite/probable stent thrombosis (%)	0	0	
Early definite/probable stent thrombosis (%)	0	0	
Late definite/probable stent thrombosis (%)	0	0	
Bleedings according to BARC classification (%)			
BARC 3,4,5	5 (1.8%)	7 (1.3%)	0.607
BARC 2	1 (0.4%)	13 (2.5%)	**0.031**
BARC 3a	2 (0.7%)	2 (0.4%)	0.517
BARC 3b	1 (0.4%)	3 (0.6%)	0.687
BARC 3c	1 (0.4%)	0	0.170
BARC 4	0	2 (0.4%)	0.304
BARC 5	1 (0.4%)	0	0.170

Abbreviations: MACE, major adverse cardiac event(s); BARC, Bleeding Academic Research Consortium; DAPT, dual antiplatelet therapy.

**Table 4:** IPTW-adjusted Kaplan–Meier estimates for primary and secondary endpoints for the entire follow-up and by landmark analysis for months 1 to 6 and months 7 to 12.

	DAPT 6 Months *n* = 339	DAPT 12 Months *n* = 645	*p* Value
MACE (%)	14.8%	16.9%	0.446
Death of any cause (%)	4.9%	4.6%	0.864
Death or myocardial infarction (%)	5.3%	5.6%	0.847
Target lesion revascularization (%)	10.8%	12%	0.607
*Months 1 to 6*			
MACE (%)	6.5%	7.1%	0.446
Death of any cause (%)	1.6%	2.2%	0.335
Death or myocardial infarction (%)	2.3%	2.7%	0.686
Target lesion revascularization (%)	3.7%	4.2%	0.576
*Months 7 to 12*			
MACE (%)	8.7%	9.9%	0.6
Death of any cause (%)	2.7%	1.7%	0.434
Death or myocardial infarction (%)	2.7%	2.3%	0.757
Target lesion revascularization (%)	6.8%	7.8%	0.633

Abbreviations: MACE, major adverse cardiac event(s); BARC, Bleeding Academic Research Consortium; DAPT, dual antiplatelet therapy.

## Data Availability

The original data presented in the study are openly available in the Harvard Dataverse at https://doi.org/10.7910/DVN/S6KDRP (accessed on 11 August 2024).

## Supplementary Materials

The following supporting information can be downloaded at: https://www.mdpi.com/article/10.3390/jcm13185449/s1, Table S1: Definition of the Bleeding Academic Research Consortium (BARC) criteria; Figure S1: Variables that were included in the Inverse Probability of Treatment Weighting (IPTW) analysis and the respective standardized mean differences before and after adjustment; Figure S2: Unadjusted (a,c,e) and IPTW-adjusted (b,d,f) time-to-event curves for the secondary endpoint death during the entire follow-up (a,b) and according to landmark analysis for months 1 to 6 (c,d) and months 7 to 12 (e,f); Figure S3: Unadjusted (a,c,e) and IPTW-adjusted (b,d,f) time-to-event curves for the secondary endpoint death or myocardial infarction during the entire follow-up (a,b) and according to landmark analysis for months 1 to 6 (c,d) and months 7 to 12 (e,f); Figure S4: Unadjusted (a,c,e) and IPTW-adjusted (b,d,f) time-to-event curves for the secondary endpoint TLR during the entire follow-up (a,b) and according to landmark analysis for months 1 to 6 (c,d) and months 7 to 12 (e,f).
