# Supplementary material for: Duration of Dual Antiplatelet Therapy after Percutaneous Coronary Intervention of Unprotected Left Main Coronary Artery Stenosis: 6 versus 12 Months
Source: J Clin Med. 2024 Sep 13;13(18):5449. doi: 10.3390/jcm13185449 (PMC11431983; doi:10.3390/jcm13185449)
Supplement: Supplementary file 1 [file jcm-13-05449-s001.zip › jcm-3169368-supplementary.pdf]

Supplement

Table S1. Definition of the Bleeding Academic Research Consortium (BARC) criteria [1]

Type of Bleeding	Summarizing Definitions of the BARC criteria
**BARC 1**	bleeding that does not require hospitalization or treatment by a healthcare professional
**BARC 2**	any overt bleeding (including bleeding found only by imaging) that does not meet the criteria for BARC 3, 4 or 5 but fulfil at least one of the following criteria:<br>(1) requiring nonsurgical, medical intervention by a healthcare professional,<br>(2) leading to hospitalization or increased level of care, or<br>(3) prompting evaluation
**BARC 3a**	overt bleeding plus haemoglobin drop of 3 to 5 g/dl,<br>any transfusion with overt bleeding
**BARC 3b**	overt bleeding plus haemoglobin drop ≥ 5 g/dl,<br>cardiac tamponade,<br>bleeding requiring surgical intervention for control<br>(excluding dental/nasal/skin/hemorrhoid),<br>bleeding requiring intravenous vasoactive agents
**BARC 3c**	intracranial hemorrhage (does include intraspinal)
**BARC 4**	CABG-related bleeding
**BARC 5**	fatal bleeding

Abbreviations: *BARC* Bleeding Academic Research Consortium, *CABG* coronary artery bypass grafting

1. Mehran, R.; Rao, S.V.; Bhatt, D.L.; Gibson, C.M.; Caixeta, A.; Eikelboom, J.; Kaul, S.; Wiviott, S.D.; Menon, V.; Nikolsky, E.; Serebruany, V.; Valgimigli, M.; Vranckx, P.; Taggart, D.; Sabik, J.F.; Cutlip, D.E.; Krucoff, M.W.; Ohman, E.M.; Steg, P.G.; White, H. Standardized bleeding definitions for cardiovascular clinical trials: a consensus report from the Bleeding Academic Research Consortium. *Circulation* **2011**, *123*, 2736-2747.

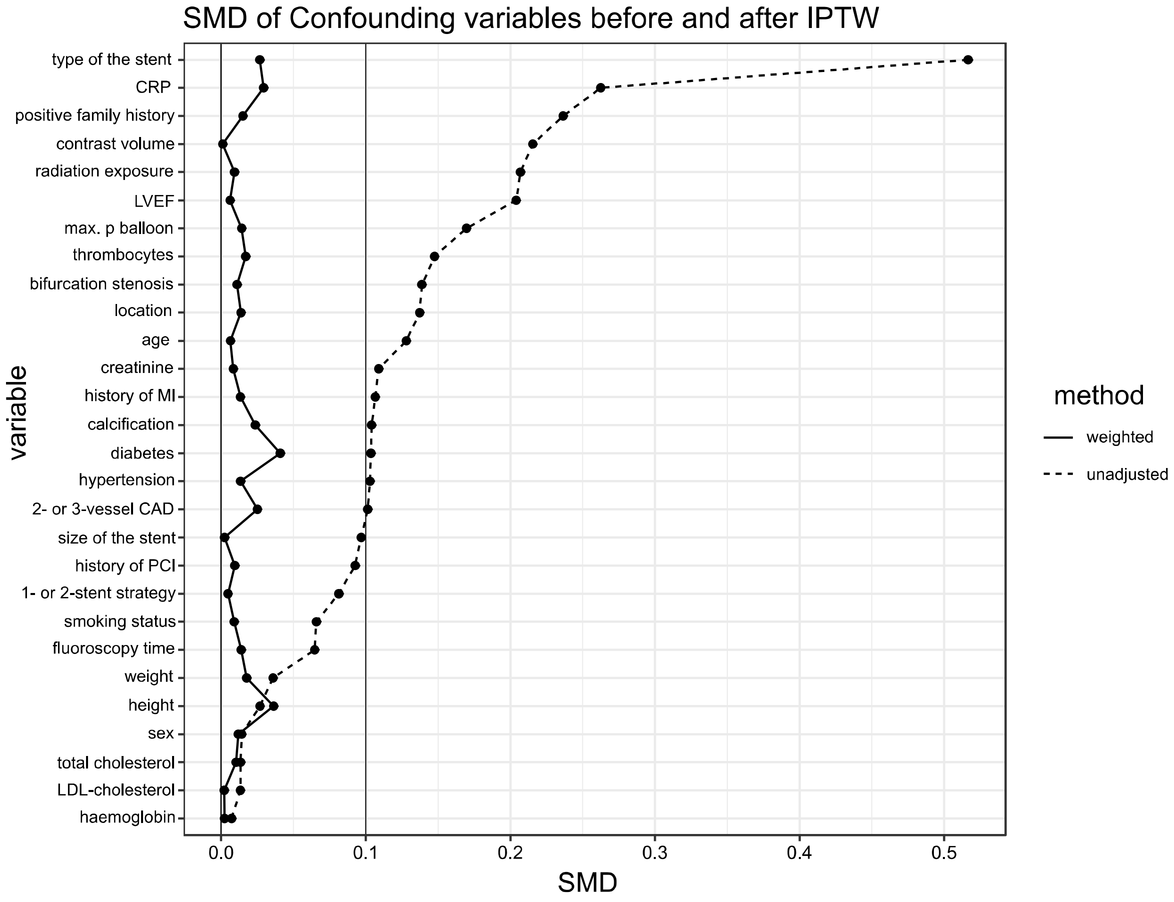

Figure S1. Variables that were included in the inverse probability of treatment weighting (IPTW) analysis and the respective standardized mean differences before and after adjustment.

Abbreviations: *SMD* standardized mean difference, *IPTW* inverse probability of treatment weighting, *CRP* c-reactive protein, *LVEF* left ventricular ejection fraction, *p* pressure, *MI* myocardial infarction, *CAD* coronary artery disease, *PCI* percutaneous coronary intervention, *LDL* low-density lipoprotein

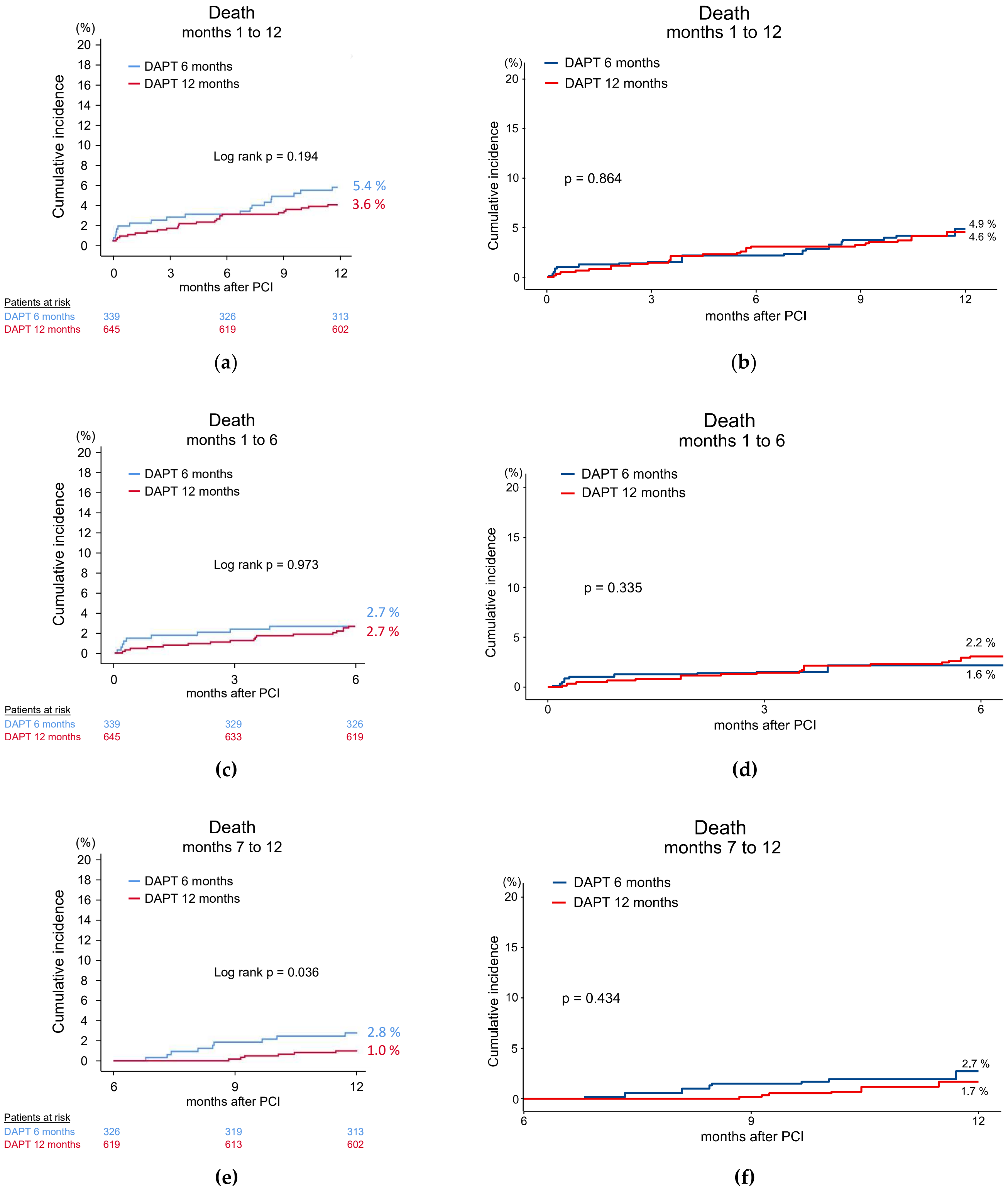

Figure S2. Unadjusted (panels a, c and e) and IPTW-adjusted (panels b, d and f) time-to-event curves for the secondary endpoint death during the entire follow-up (panels a and b) and according to landmark analysis for months 1 to 6 (panels c and d) and months 7 to 12 (panels e and f).

Abbreviations: *IPTW* inverse probability of treatment weighting, *DAPT* dual antiplatelet therapy

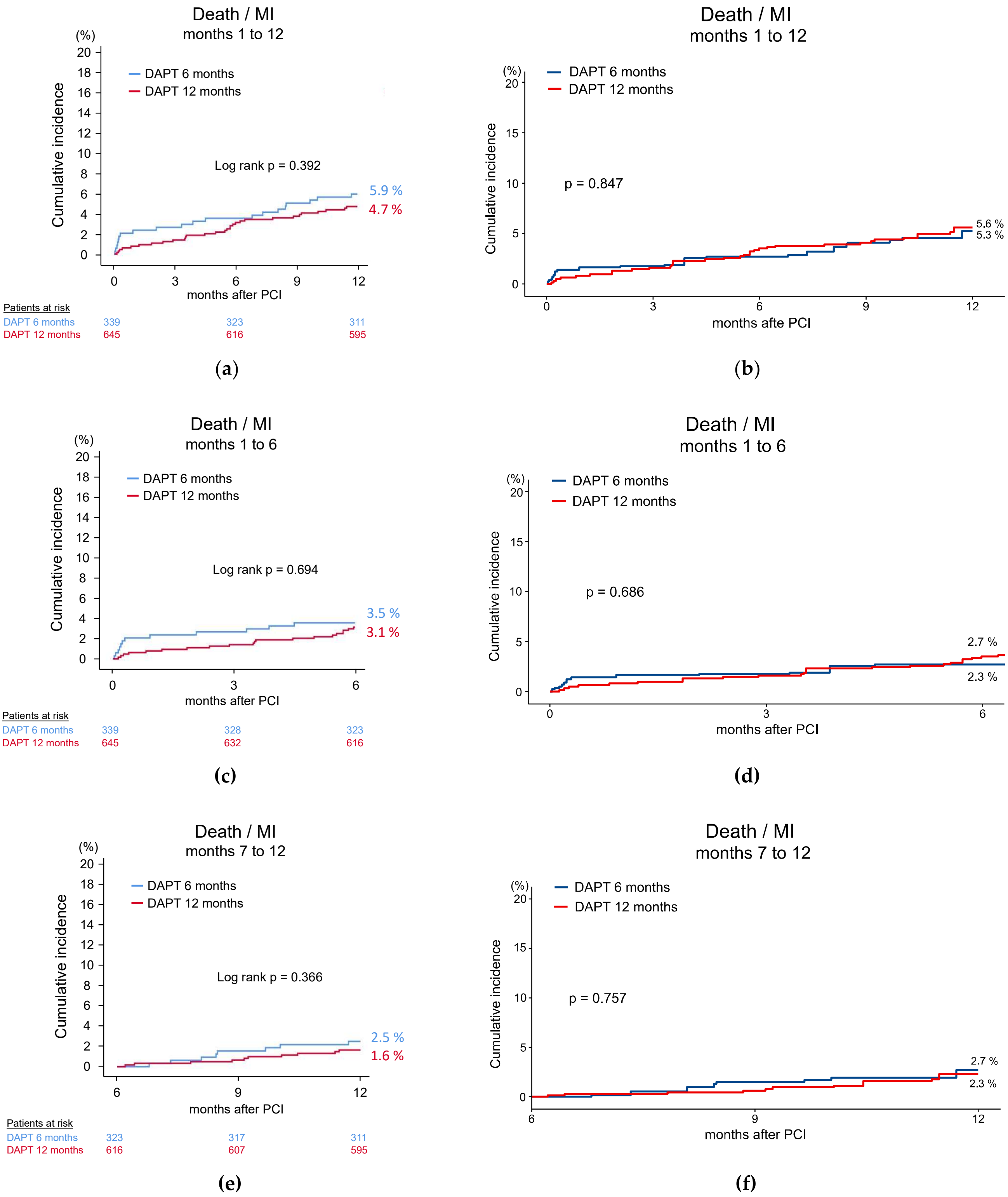

Figure S3. Unadjusted (panels a, c and e) and IPTW-adjusted (panels b, d and f) time-to-event curves for the secondary endpoint death or myocardial infarction during the entire follow-up (panels a and b) and according to landmark analysis for months 1 to 6 (panels c and d) and months 7 to 12 (panels e and f).

Abbreviations: *IPTW* inverse probability of treatment weighting, *MI* myocardial infarction, *DAPT* dual antiplatelet therapy

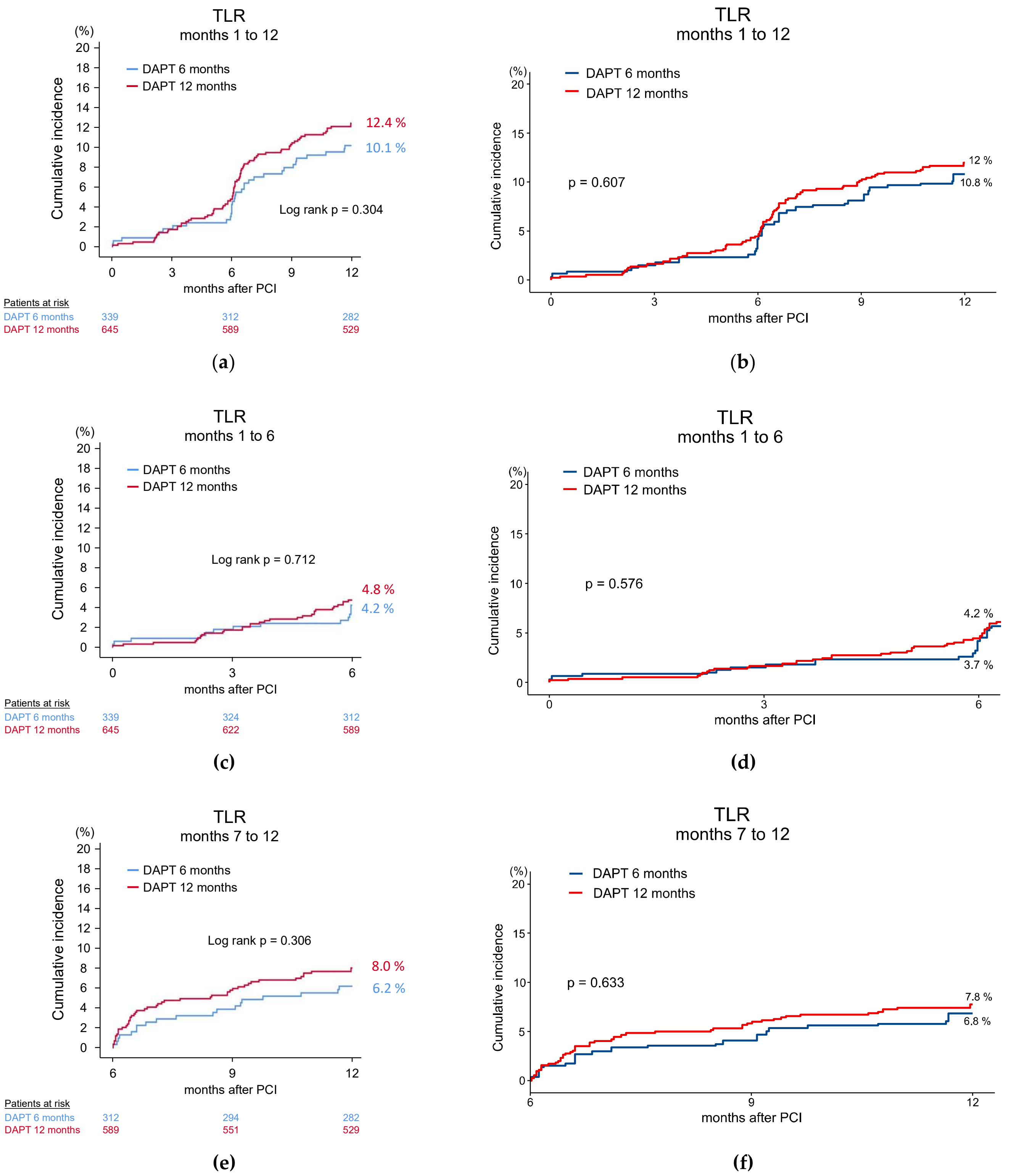

Figure S4. Unadjusted (panels a, c and e) and IPTW-adjusted (panels b, d and f) time-to-event curves for the secondary endpoint TLR during the entire follow-up (panels a and b) and according to landmark analysis for months 1 to 6 (panels c and d) and months 7 to 12 (panels e and f).

Abbreviations: *IPTW* inverse probability of treatment weighting, *TLR* target lesion revascularisation, *DAPT* dual antiplatelet therapy
